# Evidence that a West-East admixed population lived in the Tarim Basin as early as the early Bronze Age

**DOI:** 10.1186/1741-7007-8-15

**Published:** 2010-02-17

**Authors:** Chunxiang Li, Hongjie Li, Yinqiu Cui, Chengzhi Xie, Dawei Cai, Wenying Li, Victor H Mair, Zhi Xu, Quanchao Zhang, Idelisi Abuduresule, Li Jin, Hong Zhu, Hui Zhou

**Affiliations:** 1 Ancient DNA Laboratory, Research Center for Chinese Frontier Archaeology, Jilin University, Changchun 130012, PR China; 2 College of Life Science, Jilin University, Changchun 130023, PR China; 3 Xinjiang Cultural Relics and Archaeology Institute, Ürümchi 830000, PR China; 4 Department of East Asian Languages and Civilizations, University of Pennsylvania, Philadelphia, PA 19104, USA; 5 Key Laboratory of Genetic Engineering and Center for Anthropological Studies, School of Life Sciences, Fudan University, Shanghai 200433, PR China

## Abstract

**Background:**

The Tarim Basin, located on the ancient Silk Road, played a very important role in the history of human migration and cultural communications between the West and the East. However, both the exact period at which the relevant events occurred and the origins of the people in the area remain very obscure. In this paper, we present data from the analyses of both Y chromosomal and mitochondrial DNA (mtDNA) derived from human remains excavated from the Xiaohe cemetery, the oldest archeological site with human remains discovered in the Tarim Basin thus far.

**Results:**

Mitochondrial DNA analysis showed that the Xiaohe people carried both the East Eurasian haplogroup (C) and the West Eurasian haplogroups (H and K), whereas Y chromosomal DNA analysis revealed only the West Eurasian haplogroup R1a1a in the male individuals.

**Conclusion:**

Our results demonstrated that the Xiaohe people were an admixture from populations originating from both the West and the East, implying that the Tarim Basin had been occupied by an admixed population since the early Bronze Age. To our knowledge, this is the earliest genetic evidence of an admixed population settled in the Tarim Basin.

## Background

The Tarim Basin in western China, positioned at a critical site on the ancient Silk Road, has played a significant role in the history of human migration, cultural developments and communications between the East and the West. It became famous due to the discovery of many well-preserved mummies within the area. These mummies, especially the prehistoric Bronze Age 'Caucasoid' mummies, such as the 'Beauty of Loulan', have attracted extensive interest among scientists regarding who were these people and where did they come from.

Based on analyses of human remains and other archaeological materials from the ancient cemeteries (dated from approximately the Bronze Age to the Iron Age), there is now widespread acceptance that the first residents of the Tarim Basin came from the West. This was followed, in stages, by the arrival of Eastern people following the Han Dynasty [1,2]. However, the exact time when the admixture of the East and the West occurred in this area is still obscure [3]. In 2000, the Xinjiang Archaeological Institute rediscovered a very important Bronze Age site, the Xiaohe cemetery, by utilizing a device employing the global positioning system. The rediscovery of this cemetery provided an invaluable opportunity to further investigate the migrations of ancient populations in the region.

**Figure 1:**
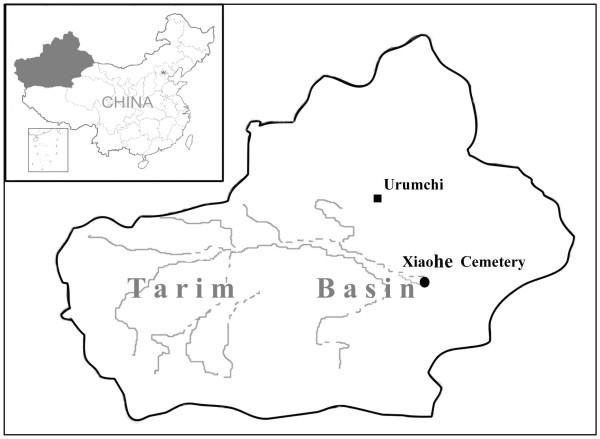
**The geographical position of Xiaohe cemetery**. The larger map shows Xinjiang, shown also in the shaded section of the map of China.

The Xiaohe cemetery (40°20'11"N, 88°40'20.3"E) is located in the Taklamakan Desert of northwest China, about 60 km south of the Peacock River and 175 km west of the ancient city of Kroraina (now Loulan; Figure 1). It was first explored in 1934 by Folke Bergman, a Swedish archaeologist, but the cemetery was lost sight of until the Xinjiang Archaeological Institute rediscovered it in 2000. The burial site comprises a total of 167 graves. Many enigmatic features of these graves, such as the pervasive use of sexual symbolism represented by tremendous numbers of huge phallus-posts and vulvae-posts, exaggerated wooden sculptures of human figures and masks, well-preserved boat coffins and mummies, a large number of textiles, ornaments and other artifacts, show that the civilization revealed at Xiaohe is different from any other archaeological site of the same period anywhere in the world [3].

The entire necropolis can be divided, based on the archeological materials, into earlier and later layers. Radiocarbon measurement (^14^C) dates the lowest layer of occupation to around 3980 ± 40 BP (personal communications; calibrated and measured by Wu Xiaohong, Head of the Laboratory of Accelerator Mass Spectrometry, Peking University), which is older than that of the Gumugou cemetery (dated to 3800). To date, these are the oldest human remains that have been excavated in the Tarim Basin [3]. A genetic study of these invaluable archeological materials will undoubtedly provide significant insights into the origins of the people of the Tarim Basin.

We examined the DNA profiles on both the maternal and the paternal aspects for all the morphologically well-preserved human remains from the lowest layer of the Xiaohe cemetery. We used these data to determine the population origins, to provide insights into the early human migration events in the Tarim Basin and, finally, to offer an expanded understanding of the human history of Eurasia.

## Methods

### Sampling

The excavation of the Xiaohe cemetery began in 2002. The lowest layer of the cemetery, comprising a total of 41 graves of which 37 have human skeletal remains, was excavated by the Xinjiang Archaeological Institute and the Research Center for Chinese Frontier Archaeology of Jilin University from 2004 to 2005. After the appropriate recording, the skeletal remains of 30 well-preserved individuals, together with sandy soil, were packed in cardboard boxes and sent to the ancient DNA laboratory of Jilin University, where they were stored in a dry and cool environment. All the samples were collected by two highly skilled scientists in our group, equipped with gloves and facemasks. As a result of the saline and alkaline character of the sand, the dry air and good drainage, the human remains are in excellent conditions. One intact femur and two tooth samples for each set of human remains were selected for DNA analysis, except for samples 84 and 121, which have only tooth samples. The archaeological information regarding the samples used in this study is summarized in Additional File 1.

### Contamination precautions and decontamination

Previous studies have shown that the recovery of authentic ancient human DNA is possible [4,5] when strict precautions are taken to prevent contamination. The following is a summary of the measures taken to avoid contamination and to ensure authenticity in the present study.

(1) Bone powdering, DNA extraction and amplification were carried out in three separate rooms in our laboratory which is dedicated solely to ancient DNA studies; all staff wore laboratory coats, facemasks and gloves and strict cleaning procedures [frequent treatment with bleach and ultraviolet (UV) light] were applied.

(2) In order to prevent contamination, polymerase chain reaction (PCR) tubes, tips, microcentrifuge tubes and drills were sterilized by autoclaving. Some of the reagents were exposed to UV light at 254 nm for at least 30 min. Tip boxes were soaked in 10% sodium hypochlorite solution. Extraction and amplification blanks were included in every PCR assay in order to detect any potential contamination from sample processing or reagents even though they were guaranteed DNA-free by the manufacturers.

(3) Multiple extractions and amplifications from the same individual were undertaken at different times and from two different parts of the skeleton by three different laboratory members.

(4) Three samples were sent to Fudan University in Shanghai, China, for an independent repetition.

(5) Cloning analysis was performed with all the samples, in order to detect potential heterogeneity in the amplification products due to contamination, DNA damage or jumping PCR.

(6) Different length fragments were amplified. Since there is an inverse correlation between fragment length and amplification efficiency for ancient DNA, contamination from modern DNA could be identified by this assay.

(7) Ancient DNA from animal remains (goat or cattle) found at the same site was isolated and amplified using the same procedures as those used for the human ancient DNA, again providing a negative control for our study.

(8) The sex of some of the morphologically intact individuals was determined by amplifying the amelogenin (AMG) gene and the results were compared to that of the morphological examination in order to monitor any potential contamination in the extraction.

(9) DNA samples from all laboratory excavators and staff members involved in the project were genetically typed and recorded for comparison with the haplotypes of all ancient samples. This is critical in order to ensure the accuracy of the generated ancient DNA results [6-9]. The information for people involved in this project is listed in Table 1.

**Table 1:** Mitochondrial DNA haplotypes of all persons involved in processing Xiaohe samples.

Investigators	Sex	HVRI polymorphism site	Appendix
Excavators			
1	Male	16189 16223 16278	
2	Male	16093 16124 16223 16311 16316	
3	Male	16223 16294 16362	
4	Male	16223 16260 16298	*
5	Male	16092 16111 16261	
6	Male	16300 16362	
7	Female	16111 16129 16266 16304	*
8	Male	16221	
9	Male	16126 16294 16296 16304	
10	Male	16085 16209 16311	
11	Male	16356	
12	Male	16136 16356	
Laboratory researchers			
1	Female	16136 16183 16189 16217 16218 16239 16248	*
2	Female	16223 16245 16362 16367	
3	Male	16183 16189 16223 16234 16290 16362	
4	Female	16126 16174 16223 16311 16362	*
5	Male	16112 16223 16362	
6	Male	16213 16223 16298 16327	
7	Male	16189 16304	

In the laboratory, the numbers 1, 2, 3 and 4 represent the staff participating in this study, 5, 6 and 7 represent the staff working in the laboratory but not directly concerned with this study. The *represents the primary researchers

### Sample preparation and DNA extraction

The bone and tooth samples were processed independently. A fragment of bone, about 3 cm long, was cut from the intact femur or tibia by sawing. In order to remove any possible surface contamination of the samples by external DNA, each bone was drilled three times with three different drills to remove a layer about 1-3 mm from the top after removing the external soil using brush, and then soaked in a 5% sodium hypochlorite solution for 10 min, rinsed with distilled water and absolute alcohol successively and UV-irradiated (254 nm) on all sides for at least 45 min in a clean room. The samples were then pulverized by Freezer Mill 6850 after immersion in liquid nitrogen. Intact teeth were handled differently: they were soaked in a 5% sodium hypochlorite solution for 15 min after a preliminary treatment in which they were wiped with 10% sodium hypochlorite and then rinsed with absolute alcohol. After that, all sides of the samples were exposed to UV radiation (254 nm) for a minimum of 20 min before the sample was ground to powder by Freezer Mill 6750. DNA was extracted by means of a silica-based protocol [10]. One extraction blank was included for every three ancient samples. In brief, 0.5-2 g tooth/bone powder was incubated about 20 h at 50°C in lysis solution (1 mL 10% SDS, 4 ml EDTA (pH 8; 0.5 M, Promega, WI, USA), 100 uL of 10 mg/ml proteinase K (Merck, Darmstadt, Germany). After centrifugation, the solution was subsequently concentrated with centricons (Millipore, MA, USA) up to about 100 μL volume, and then the extraction proceeded according to the handbook of the QIAquick DNA Purification Kit (Qiagen, Hilden, Germany).

### DNA amplification, cloning, and sequencing

The nucleotide positions 16035-16409 of the mitochondrial genome was amplified by two overlapping primer pairs. In addition, a number of coding-region mtDNA polymorphisms were typed, which are diagnostic for major branches in the mtDNA tree: Haplogroups R(12705C), UK(12308G), HV(14766T), H(7028C), R1(4917G), R11(10031C) and C4(11969A) were identified by direct sequencing and haplogroups M(10400T), F(3970T) and C(14318C) were examined by using amplified product-length polymorphisms method [11-13]. The B haplogroup was identified based on 9-bp deletion in np8280. Some Y-chromosomal single nucleotide polymorphisms (Y-SNPs) were typed, which are diagnostic for major branches in the Y chromosome haplogroup tree [14,15]: Haplogroups F(M89T), K(M9G), P(M45A), R1(M173A), and R1a1a(M198A) were identified by direct sequencing. The primers used in HVRI and diagnostic SNP markers are shown in Table 2.

**Table 2:** The primers used in this study.

Haplogroup/AMG	Primer	SNP	Length
HVRI-AB	L16017 5'-TTCTCTGTTCTTTCATGGGGA H16251 5'-GGAGTTGCAGTTGATGTGTGA	Sequencing	235 bp
HVRI-CD	L16201 5'-CAAGCAAGTACAGCAATCAAC H16409 5'-AGGATGGTGGTCAAGGGA	Sequencing	209 bp
M	10400T 5'-taattaTACAAAAAGGATTAGACTGtgCT 10400C 5'-TACAAAAAGGATTAGACaGAACC 10400R 5'-GAAGTGAGATGGTAAATGCTAG	10400T	149 bp/142 bp
R	L12604 5'-ATCCCTGTAGCATTGTTCG H12754 5'-GTTGGAATAGGTTGTTAGCG	12705C	151 bp
UK	L12247 5'-TAACAACATGGCTTTCTCAACT H12377 5'-GAAGTCAGGGTTAGGGTGGT	12308G	132 bp
C	L14318T 5'-CCTTCATAAATTATTCAGCTTCCaACACTAT L14318C 5'-aaaaagctaCATAAATTATTCAGCTTCCTACtCTAC H14318R 5'-TTAGTGGGGTTAGCGATGGA	14318C	110 bp/115 bp
C4	L11845 5'- AAGCCTCGCTAACCTCGCC H12120 5'- GGGTGAGTGAGCCCCATTG	11969A	176 bp
B	L8215 5' ACAGTTTCATGCCCATCGTC ' H8297 5' ATGCTAAGTTAGCTTTACAG	CoII/tRNAlys 9-bp deletion	121 bp/112 bp
R1	L4812 5'- GTCCCAGAGGTTACCCAAG H4975 5'- CCACCTCAACTGCCTGCTA	4917G	164 bp
R11	L9920 5'- CGCCTGATACTGGCATTTTGT H101075' -GTAGTAAGGCTAGGAGGGTGTTG	10031C	188 bp
HV	L14668 5'- CATCATTATTCTCGCACGG H14831 5'- CGGAGATGTTGGATGGGGT	14766T	164 bp
F	3970T 5' taaaaTGTATTCGGCTATGAAGAtTAA 3970C 5' GTGTATTCGGCTATGAAGtATAG 3970R 5' AGTCTCAGGCTTCAACATCG	3970T	70 bp/66 bp
H	L6966 5'-GGCATTGTATTAGCAAACTCAT H7118 5'-TAGGGTGTAGCCTGAGAATAG	7028C	152 bp
AMG	AMG1 5'-CCTGGGCTCTGTAAAGAATAG AMG2 5'-CAGAGCTTAAACTGGGAAGCTG		115 bp/121 bp
Paternal hg F	Forward 5' CCACAGAAGGATGCTGCTCA Reverse r 5' CACACTTTGGGTCCAGGATCAC	M89T	125 bp
Paternal hg K	Forward 5' GGACCCTGAAATACAGAAC Reverse 5' AAGCGCTACCTTACTTACAT	M9G	128 bp
Paternal hg P	Forward 5' GGGTGTGGACTTTACGAAC Reverse 5' AAATCCTACTATCTCCTGGC	M45A	129 bp
Paternal hg R1	Forward 5' TTACTGTAACTTCCTAGAAAATTGG Reverse 5' ATCCTGAAAACAAAACACTGG	M173C	126 bp
Paternal hg R1a1a	Forward 5' CTCTTTAAGCCATTCCAGTCA Reverse 5' AAACATTACATGAGAAATTGCTG	M198A	113 bp

PCR amplifications were performed in 20 μL reactions with 3 μl of extract, 1 U Taq DNA polymerase (Fermentas, Ontario, Canada), and 1.5×buffer (Fermentas), 1.5 mg/ml BSA, 2.5 mM MgCl2, 0.2 mM dNTP (Promega, USA), and 400 pmol for each primer (Sangong, China). The cycle conditions used a Mastercycler gradient (Eppendorf, Germany) consisting of 40 cycles at 94°C for 1 min, 59°C -52°C for 1 min, and 72°C for 1 min, with a first denatured step of 94°C for 5 min and a last extended step of 72°C for 10 min. PCR products were purified with QIAamp quick DNA Purification Kit (Qiagen) and sequenced with BigDye 3.1 in an ABI 310 DNA sequencer (Applied Biosystems, CA, USA). The PCR products were cloned with pGEM-T (Promega), following the supplier's instructions. Extraction, amplification, cloning, and sequencing were undertaken in slightly varying conditions for different samples (Table 3).

**Table 3:** Analysis strategy of the samples.

Sample	MtDNA-HVRI	MtDNA	Y chromosome	Sexing		Independent
					
No.	haplotype	haplogroup	haplogroup	Morphological	Molecular	repetition
100	298-327	C4		Female	Female	√
102	298-327	C4		Female	-	
106	298-327	C4	R1a1a	Male	Male	
107	223-298-309-327	C4		Female	-	
109	298-327	C4		Female	-	
110	298-327	C4		Female	-	
111	223-298-309-327	C4	R1a1a	Male	Male	
115	298-327	C4	R1a1a	Male	Male	
117	223-304	M*		Female	Female	
119	93-134-224-311-390	K		Female	Female	√
120	189-192-311	R*	R1a1a	Male	Male	
121	183-189-192-311	R*	R1a1a	Male	Male	
127	223-298-309-327	C4		Female	Female	√
128	260	H		Female	Female	
131	189-192-311-390	R*		Female	Female	
132	298-327	C4		Female	Female	
135	223-298-309-327	C4		Female	Female	
136	298-327	C4	R1a1a	Male	Male	
138	298-327	C4	-	Female	-	
139	298-327	C4	R1a1a	Male	Male	

Note: √ means we performed this kind of strategy to the sample; - indicates that the targeted DNA fragment could not be amplified for a given sample.

### Data analysis

Sequence alignments were performed using the Clustalx1.8 software. Comparison of DNA sequence homology was performed with Blast from the National Centre for Biotechnology Information. Median networks were constructed by Network 4.5 using a reduction threshold and the different weights for SNPs loci but the same weight for all HVSI polymorphism loci. No statistical analysis was performed in this study due to the small sample size.

## Results

### Authenticity of results

A total of 23 reproducible mtDNA fragments (360 bp) were obtained from 30 individual sets of the Xiaohe human remains, after discarding seven samples due to failed amplification or irreproducible results. Three of the 23 DNA fragments were similar to the DNA of two people involved in the study and were also removed from the study, even though they yielded consistent results through three or more independent extractions.

The remains of 20 individuals were selected for cloning analysis. Eight to twelve clones from two independent amplifications of the 20 individuals were selected for automated DNA sequencing. Although a few positions were different from direct sequencing of the PCR products, which could be due to random Taq misincorporation or DNA damage, the consensus sequence from cloning was consistent (see Additional File 2). In order to validate the results generated in our laboratory, human remains from three Xiaohe individuals (100, 119, and 127) were sent to the ancient DNA laboratory of Fudan University for further analysis and identical results were obtained.

The following facts further confirm the authenticity of our results: (1) an inverse correlation between the size of the PCR amplicons and the amplification efficiency for the Xiaohe samples (209 bp > 235 bp > 399 bp) was found; (2) molecular sexual identification results were in accordance with morphological sex assignments; (3) HVR-I sequences match well with the key coding region SNPs according to the well-defined mtDNA phylogenetic tree [16,17]; and (4) parallel studies were also performed on Xiaohe animal samples, and no human DNA was detected. Collectively, 20 mtDNA fragments from 30 human remains were obtained that were inferred to be authentic (Table 3). The sequences have been submitted to GenBank with accession numbers FJ719792-FJ719811. This high success rate suggests that the DNA from the Xiaohe human remains is well preserved, as would be expected in samples originated from dry environmental conditions.

### MtDNA haplogroup profile and distribution

**Figure 2:**
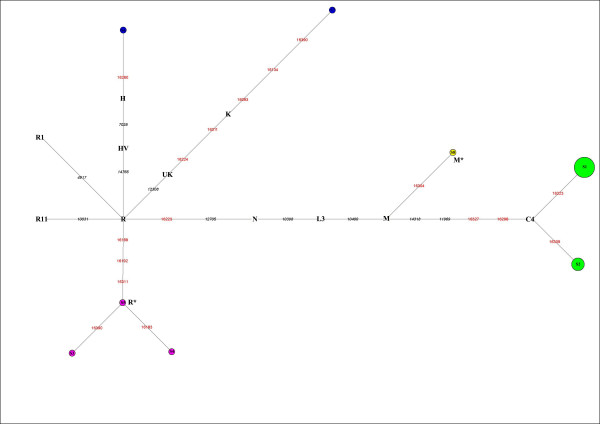
**Reduced median network of Xiaohe sequences**. Node size is proportional to frequencies. HVR1 positions are numbered relative to Cambridge reference sequence (CRS). Single nucleotide polymorphism diagnostic positions are in black italics; green represents East Eurasian lineage C, containing 14 individuals. Xiaohe R* is the cluster under the macrohaplogroup R.

The 20 mtDNA fragments containing eight different haplotypes that can be further assigned to five haplogroups, which belong to subhaplogroups of macrohaplogroups M and N (Figure 2).

The dominant haplogroup in the Xiaohe people was the East Eurasian lineage C, shared by 14 Xiaohe individuals who were associated with two different mtDNA haplotypes (S1 and S2). According to the coding region 11969 G to A, all lineage C found in the Xiaohe people was further changed to subhaplogroup C4, which had D-loop group-specific polymorphisms at nucleotide positions (np) 16298 (T to C) and 16327 (C to T) [18]. Interestingly, the haplotype S1 shared by these 10 of the 14 Xiaohe individuals did not have the 16223T, a mutation had been found in the majority of modern lineage C populations. This haplotype was found merely in modern Evenks and Udegeys of southeastern Siberia but the frequency is low [18]. It is interesting to note that our study found a mutation, unique to the Xiaohe people but rare among lineage C, 16309 G (see Additional File 3). To the best of our knowledge, the lineage C with 16309 G was observed only in three people from modern central Asia to date, who also possess another two mutations in HVRI region [19].

Besides the East Eurasian lineage, two West Eurasian mtDNA haplogroups H and K were found among the Xiaohe people. H lineage is the most common mtDNA haplogroup in West Eurasia [20], but haplogroup H with a 16260T was shared by only nine modern people in Genbank, including one Italian, one German, one Hungarian, one Portuguese, one Icelander and four English people. Haplogroup K, a western Eurasian-specific haplogroup, is mainly distributed in Europe, central Asia, and Iran [20,21]. However, haplogroup K with 16134T, found in the Xiaohe people, has not been found in modern people to our knowledge.

Among the Xiaohe people, three sequences with the unique HVRI motif 16189-16192-16311 formed a subcluster (Figure 2) and were not shared by modern people. They are identified as macrohaplogroup R through sequencing the PCR amplicons at np10400 and np12705 in the coding region. The np12308, np14766, np10031, np4917, np3970, and 9 bp deletion, which are the diagnostic sites for the main subhaplogroups of R, were further examined [15]. The results showed that they are related neither to the West Eurasian haplogroups UK, TJ, HV, R11 and R1, nor to the East Eurasian haplogroups B and F. So we designated them as haplogroup R* temporarily. Another sequence with motif 16223-16304, shared by some people from East Asia, India, and Europe, was assigned to haplogroup M*.

### Y chromosome haplogroup profiling and distribution

Fifteen individuals' AMG amplicons were obtained from the 20 Xiaohe individuals (whose mtDNA was successfully amplified), among which seven individuals were identified as male and eight as female. The Y chromosome haplogroup of the seven males were all assigned to haplogroup R1a1a through screening the Y-SNPs at M89, M9, M45, M173 and M198 successively. Haplogroup R1a1a is widely distributed in Eurasia: it is mainly found in Eastern Europe, Central Asia, South Asia, Siberia, ancient Siberia, but rare in East Asia [22-24].

## Discussion

The Xiaohe cemetery is the oldest archeological site with human remains discovered in the Tarim Basin to date. Our genetic analyses revealed that the maternal lineages of the Xiaohe people were originated from both the East and the West, whereas paternal lineages discovered in the Xiaohe people all originated from the West.

**Figure 3:**
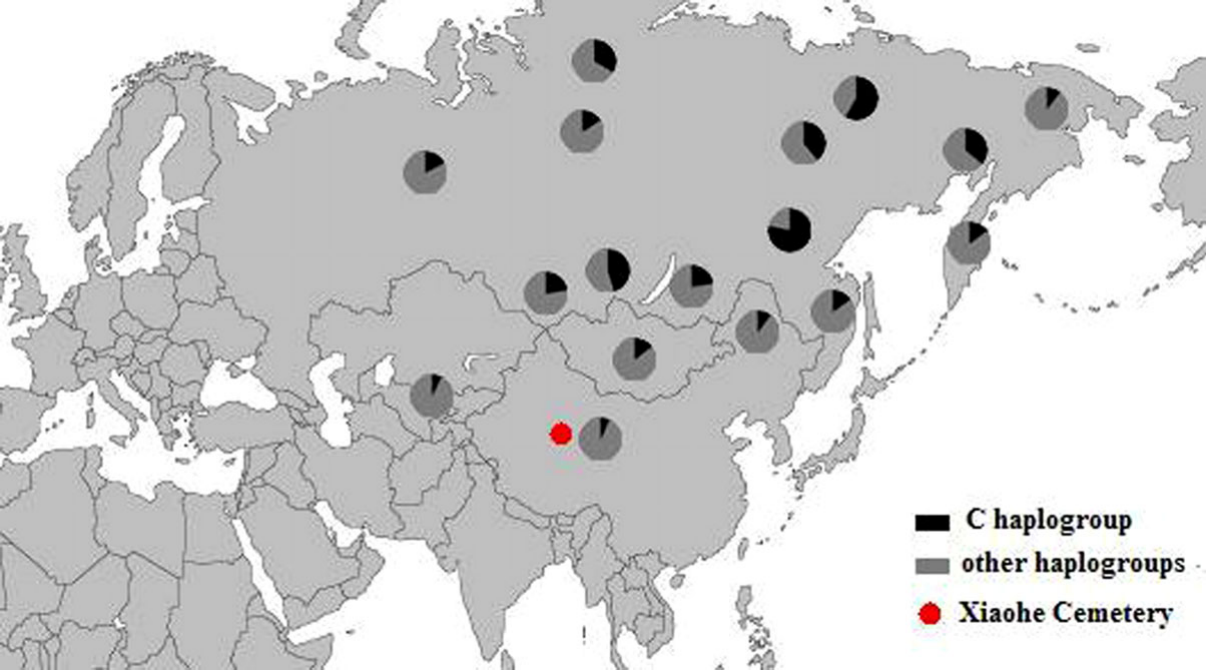
**Map of Eurasia, showing the approximate distribution of haplogroup C**. Black sections reflect the frequency of haplogroup C data taken from references listed in Additional File 3.

**Figure 4:**
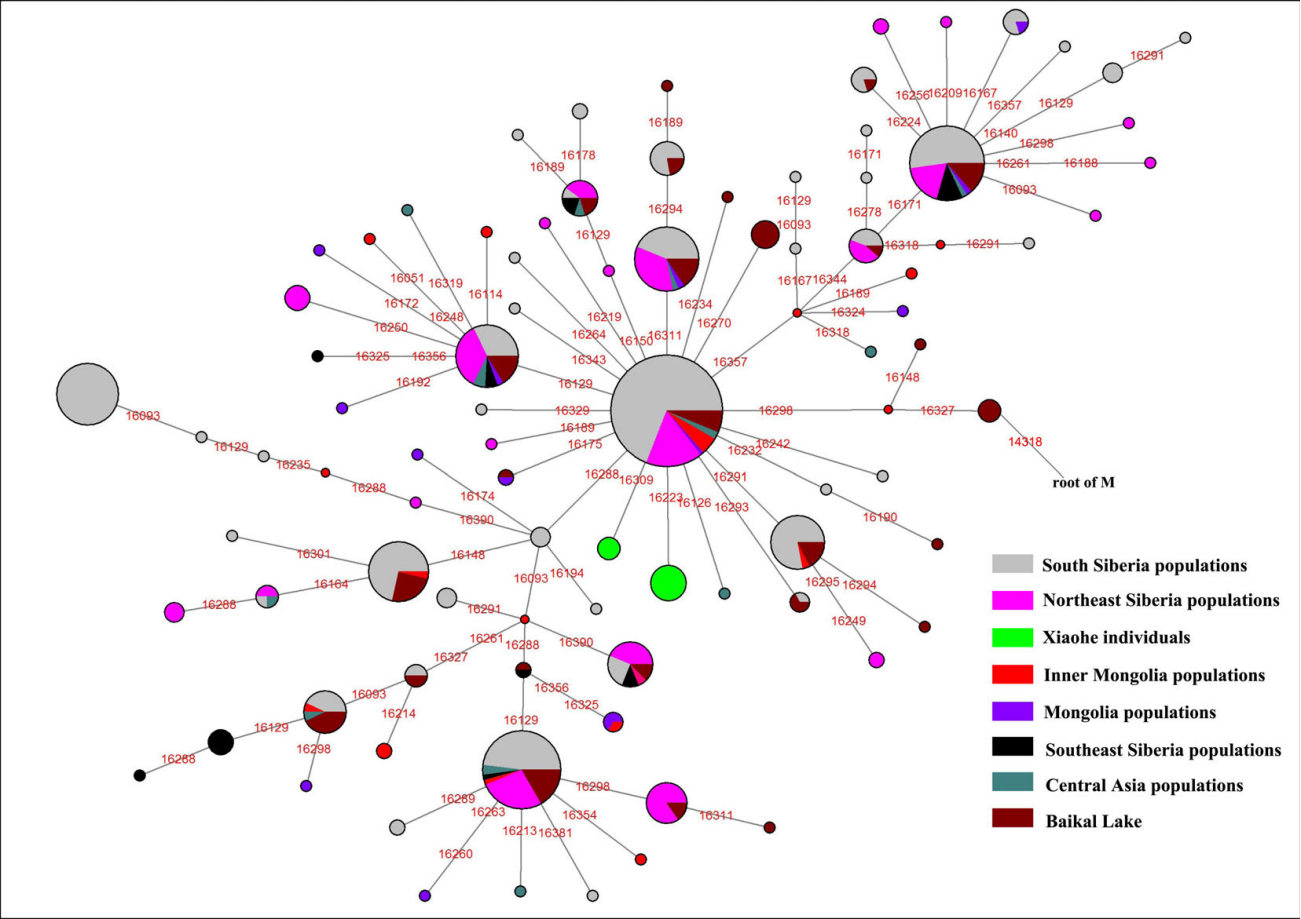
**Phylogenetic tree of haplogroup C, based on HVS-I sequences in the region 16050-16391**. For references for the mitochondrial DNA sequences in this study see Additional File 3; the length difference mutations were excluded from this analysis.

The East Eurasian lineage C, which was widely distributed in modern Asian populations, was the dominant haplogroup in the remains recovered from the lowest layer of the Xiaohe cemetery. This lineage is most frequently found in modern Siberian populations (Evenks, Yakut, Evens, Tuvinian, Buryat, Koryak and Chukchi) and to a lesser extent in modern East Asian (Mongolian, Daur and Korean) and Central Asian populations [25-29] (Figure 3). It was also found in the ancient Qinghai (4000BP) of China [30] and ancient South Siberian populations [31,32]. In order to trace the original wellspring of lineage C in the Xiaohe population, a phylogenetic tree was constructed using 14 ancient Xiaohe samples and 522 modern haplogroup C samples from surrounding areas of the Xiaohe cemetery, including Siberian, Mongolian, Central Asian, northern Chinese and northern minorities of East Asia (see Additional File 3). The phylogenetic network displays a star-like distribution within the South Siberian population, which has an ancestral haplotype motif 16223-16298-16327. The ancestral haplotype was found mainly in South Siberian whose diversity of haplotypes C is very high (Figure 4). Therefore, the original source of haplogroup C was inferred to South Siberian. It is important to note that the C haplotypes of the Xiaohe people had only a single mutation compared with the ancestral haplotype. The shared sequences of the Xiaohe C haplotype (S1) were distributed in southeastern Siberia. It implies that the east Eurasian component in the Xiaohe people originated from the Siberian populations, especially the southern or eastern Siberian populations.

The mtDNA haplogroup H is the most common mtDNA haplogroup in Europe, especially in northwestern Europe, and its frequency can be as high as 65% in Iberia. Frequencies gradually decrease from the northwest to the southeast of Europe. By contrast, the frequency of haplogroup H rises to only 20% in the Near East, and to less than10% in Central Asia, and is very low in East Asia [33,34]. All of the shared sequences of the Xiaohe H haplotype, however, were distributed in Western Europe. Haplogroup K is also common in Europe, particularly around the Alps and the British Isles. It is found with less frequency in North Africa, the Middle East, and South Asia [21,35-37]. Considering the presence of haplogroups H and K in the Xiaohe people and the geographical distribution of shared sequences, we conclude that the west Eurasian component observed in the Xiaohe people originated from western European, and maternal ancestry of the Xiaohe people might have close relationship with western European.

Regarding the Y chromosomal DNA analyses, the seven males identified all belonged to haplogroup R1a1a. It is most frequently found in Eastern Europe, South Asia and Siberia. In contrast, it is relatively uncommon in Middle Easterners and rare in East Asian [22-24]. It is thought to be a trace of the migration events of early Indo-European [38,39]. The presence of haplogroup R1a1a in the ancient Xiaohe people implies that the parental ancestry of the Xiaohe people originated from somewhere in Siberia or Europe, which is consistent with the origin of maternal ancestry.

It is generally agreed that the origin of modern populations in Xinjiang and Central Asia is the result of the admixture of people from the West and the East [19,25,40]. When and where this admixture first occurred has long been of interest to geneticists and archaeologists [41-44]. The year 132 BC is often considered to be the beginning of contact between the East and the West along the Great Silk Road, since the Chinese explorer Zhang Qian went westward into Central Asia at that time. However, Mair has suggested that the date should be even earlier, based on the fact that silk appeared in Europe at 1000 BC [1]. In this study, the East and West Eurasian lineages are seen to coexist in the Xiaohe people, implying that the East had contacted the West during the early Bronze Age. It is noteworthy that the maternal lineage of five male individuals (106, 111, 115, 136 and 139) originated from East Eurasian, whereas their paternal lineage originated from the West Eurasian, implying that the Xiaohe population had been an admixture of people from both the West and the East. Given the unique genetic haplotypes and the particular archaeological culture, the time of this admixture could be much earlier than the time at which the Xiaohe people were living at the site. This means that the time of their mingling was at least a 1000 years earlier than previously proposed.

However, the mtDNA haplogroups H, K and C all are very ancient lineages, over 10,000 years old in vast north Eurasia, whereas the civilization of the Tarim Basin, according to the archaeological materials, arose very late. The admixture therefore probably occurred elsewhere, before immigration into the Tarim Basin. The Xiaohe people might well have been an admixture at the time of their arrival. Where did the initial admixture occur?

**Figure 5:**
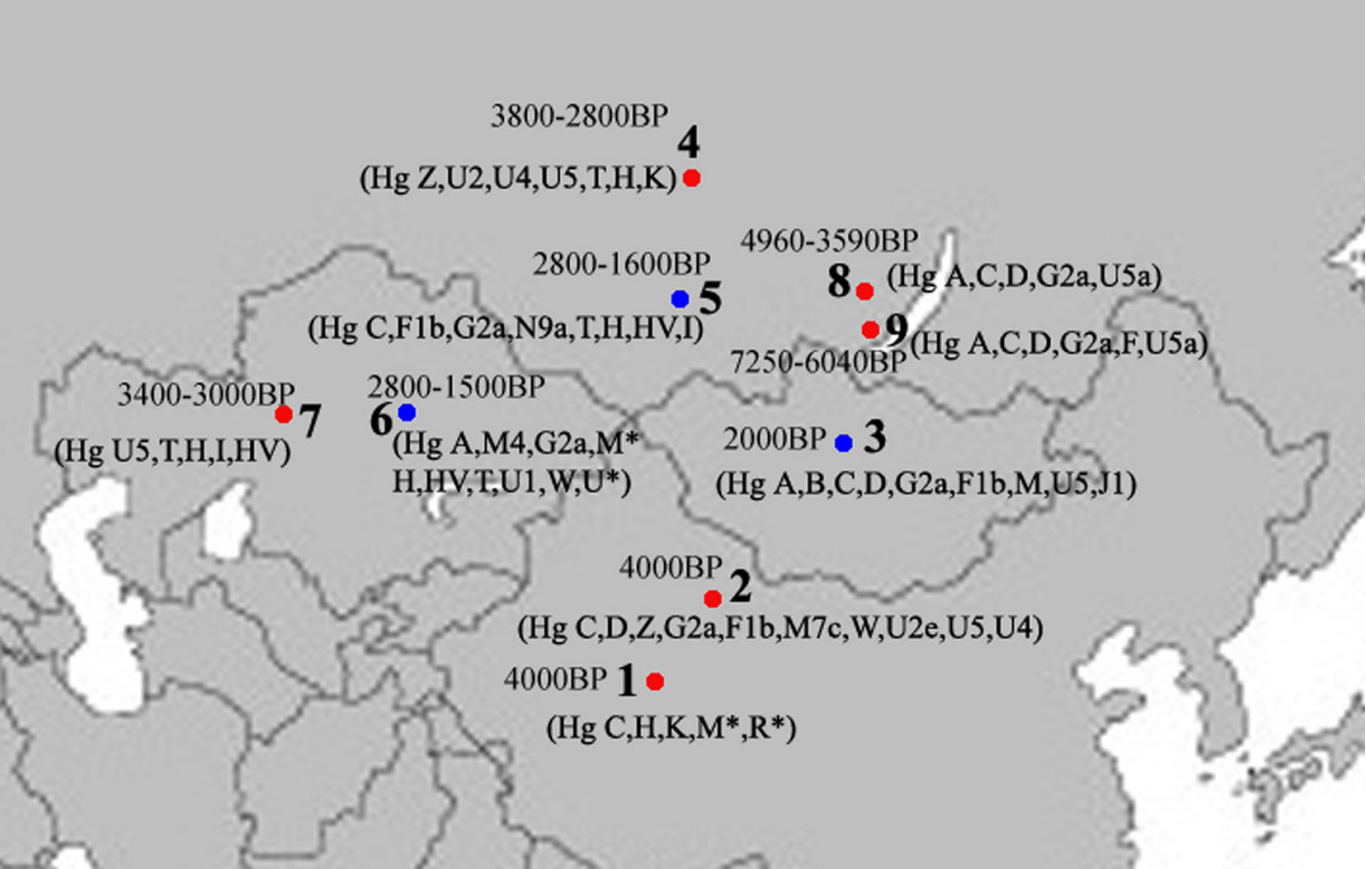
**Map of Eurasia, showing ancient populations from the Tarim Basin and surroundings**. Number 1 represents Xiaohe cemetery, data from this study; number 2 represents Xinjing Hami cemetery, data not published; Number 3 represents ancient Xiongnu, data from reference 46; Numbers 4 and 5 represent ancient South Siberian people, data from reference 38, Numbers 6 and 7 represent ancient Central Asians, data from reference 41; Numbers 8 and 9 represent ancient Lake Baikal people, data from reference 45. The red colour represents that the data was generated from samples from about Bronze Age and/or the prehistory era, while blue represents that the data was generated from samples from Iron Age.

The admixture from people of the West and the East was also found in ancient Central Asia, Siberia, and Mongolia [16,38,45,46]. The extent of the admixture varied in different regions and at different periods (Figure 5). Central Asia has always been the crossroads of contact between the West and the East. Lalueza-Fox *et al*. proved that Eastern lineages coexisted with Western lineages in Central Asia after 700 BC [41], whereas the West had met the East in south Siberia in the Bronze Age [38], and even earlier at Lake Baikal [45]. Xinjiang and the surrounding areas, especially south Siberia, were places at which the contact between western and eastern populations occurred earlier than in Central Asia. Given the fact that the mtDNA haplogroup C was distributed mainly in south Siberia, and that haplogroups H, K and R1a1a already had spread eastward into south Siberia during the Bronze Age, it is possible that the initial admixture occurred somewhere in southern Siberia. Considering that the cultural characteristics of the Xiaohe cemetery are similar to those of the Andronovo or Afanasevo culture that appeared throughout the southern Russian steppe, Kazakhstan, and western Central Asia during the second millennium BC [1,46], the admixed population might have had relationship with populations settled South Siberia during the Bronze Age.

## Conclusions

Our results demonstrated that the Xiaohe people was an admixture from populations originating from both the West and the East, implying that the Tarim Basin had been occupied by an admixed population since the early Bronze Age. Considering the unique genetic haplotypes and particular archaeological culture, the admixed population might have had relationship with populations settled South Siberia during the Bronze Age. To our knowledge, this is the earliest genetic evidence of an admixed population settled in the Tarim Basin.

## Abbreviations

PCR: polymerase chain reaction; mtDNA: mitochondrial DNA: SNP: single nucleotide polymorphism; CRS: Cambridge reference sequence.

## Authors' contributions

CXL and HJL contributed equally to this work, they performed the molecular genetic studies and data analysis and wrote the manuscript. YQC and VHM helped to draft the manuscript. CZX and DWC participated in performing experiments. WYL and IA provided materials and background documents. QCZ participated in the statistical analysis. ZX and LJ provided independent replication. HZ participated in conceiving and designing the study. HZ designed the study and wrote the manuscript. All authors read and approved the final manuscript.

## Supplementary Material

Additional file 1 **Table S1**. Archaeological information for 30 Xiaohe individuals. Click here for file

Additional file 2 **Figure A1**. The results of clone sequencing. Click here for file

Additional file 3 **Table S2**. Estimated frequencies of mitochondrial DNA haplogroup C in modern populations. Click here for file
