# Supplementary material for: Evidence that a West-East admixed population lived in the Tarim Basin as early as the early Bronze Age
Source: BMC Biol. 2010 Feb 17;8:15. doi: 10.1186/1741-7007-8-15 (PMC2838831; doi:10.1186/1741-7007-8-15)
Supplement: Additional file 1 — Table S1. Archaeological information for 30 Xiaohe individuals. [file 1741-7007-8-15-S1.DOC]

| individuals | Sex | Age | Hair colour | sampling |
| --- | --- | --- | --- | --- |
| 84 | female | adult | Black hair | teeth |
| 85 | female | adult |  | teeth/bone |
| 93 | female | 35-40 | nut-brown hair | teeth/bone |
| 100 | female | adult |  | Teeth/bone |
| 102 | female | 25-30 |  | teeth/bone |
| 104 | female | 45± | brown-black hair | teeth/bone |
| 106 | male | 35**±** |  | teeth/bone |
| 107 | female | 40-45 | nut-brown hair | teeth/bone |
| 108 | female |  |  | teeth/bone |
| 109 | female | 50**±** | Light brown hair | teeth/bone |
| 110 | female | 19-20 | brown hair | teeth/bone |
| 111 | male | 25-30 | brown hair | teeth/bone |
| 112 | female | 25-30 | brown hair | teeth/bone |
| 114 | male |  |  | teeth/bone |
| 115 | male | 35-40 | Brown black hair | teeth/bone |
| 117 | female | 40± |  | teeth/bone |
| 119 | female | 30-35 | Light brown hair | teeth/bone |
| 120 | male | 45**±** | Brown black hair | teeth/bone |
| 121 | male | adult |  | teeth |
| 125 | female | adult | flaxen hair | teeth/bone |
| 127 | female | 55**±** |  | teeth/bone |
| 128 | female | 40± | Brown black hair | teeth/bone |
| 131 | female | 30-35 | nut-brown hair | teeth/bone |
| 132 | female | 40**±** | nut-brown hair | teeth/bone |
| 134 | ? | 14-15 | Brown black hair | teeth/bone |
| 135 | female | 50**±** | black hair | teeth/bone |
| 136 | male | 35**±** | nut-brown hair | teeth/bone |
| 137 | female | 45-50 |  | teeth/bone |
| 138 | female | 35**±** | Brown black hair | teeth/bone |
| 139 | male | 40-45 | Brown hair | teeth/bone |

Table S1 Archaeological information for 30 Xiaohe individuals.
