# Supplementary material for: Evidence that a West-East admixed population lived in the Tarim Basin as early as the early Bronze Age
Source: BMC Biol. 2010 Feb 17;8:15. doi: 10.1186/1741-7007-8-15 (PMC2838831; doi:10.1186/1741-7007-8-15)
Supplement: Additional file 2 — Figure A1. The results of clone sequencing. [file 1741-7007-8-15-S2.PDF]

```
       1111 1111111111 1111111111 1111111111 1111111111 1111111111 1111111111 1111111111 1111111111 1111111111 1111111111 1111111111 1111111111 1111111111 1111111111 1111111111 1111111111 1111111111 1111111111 1111111111 1111111111 1111111111 1111111111 1111111111 1
       6666 6666666666 6666666666 6666666666 6666666666 6666666666 6666666666 6666666666 6666666666 6666666666 6666666666 6666666666 6666666666 6666666666 6666666666 6666666666 6666666666 6666666666 6666666666 6666666666 6666666666 6666666666 6666666666 6666666666 6
       0000 0000000000 0000000000 0000000000 0000000000 0000000000 0000000000 0000000000 0000000001 1111111111 1111111111 1111111111 1111111111 1111111111 1111111111 1111111111 1111111111 1111111111 1111111112 2222222222 2222222222 2222222222 2222222222 2222222222 2
       1112 2222222223 3333333334 4444444445 5555555556 6666666667 7777777778 8888888889 9999999990 0000000001 1111111112 2222222223 3333333334 4444444445 5555555556 6666666667 7777777778 8888888889 9999999990 0000000001 1111111112 2222222223 3333333334 4444444445 5
       7890 1234567890 1234567890 1234567890 1234567890 1234567890 1234567890 1234567890 1234567890 1234567890 1234567890 1234567890 1234567890 1234567890 1234567890 1234567890 1234567890 1234567890 1234567890 1234567890 1234567890 1234567890 1234567890 1234567890 1
CRS    TTCT CTGTTCTTTC ATGGGGAAGC AGATTTGGGT ACCACCCAAG TATTGACTCA CCCATCAACA ACCGCTATGT ATTTCGTACA TTACTGCCAG CCACCATGAA TATTGTACGG TACCATAAAT ACTTGACCAC CTGTAGTACA TAAAAACCCA ATCCACATCA AAACCCCCTC CCCATGCTTA CAAGCAAGTA CAGCAATCAA CCCTCAACTA TCACACATCA ACTGCAACTC C
100AB1 .... .......... .......... .......... .......... .......... .......... .......... .......... .......... .......... .......... .......... .......... .......... .......... .......... .......... .......... .......... .......... .......... .......... .......... .
100AB2 .... .......... .......... .......... .......... .......... .......... .......... .......... .......... .......... .......... .......... .......... .......... .......... .......... .......... .......... .......... .......... .......... .......... .......... .
100AB3 .... .......... .......... .......... .......... .......... .......... .......... .......... .......... .......... .......... .......... .......... .......... .......... .......... .......... .......... .......... .......... .......... .......... .......... .
100AB4 .... .......... .......... .......... .......... .......... .......... .......... .......... .......... .......... .......... .......... .......... .......... .......... .......... .......... .......... .......... .......... .......... .......... .......... .
100AB5 .... .......... .......... .......... .......... .......... .......... .......... .......... .......... .......... .......... .......... .......... .......... .......... .......... .......... .......... .......... .......... .......... .......... .......... .
100AB6 .... .......... .......... .......... .......... .......... .......... .......... .......... .......... .......... .......... .......... .......... .......... .......... .......... .......... .......... .......... .......... .......... .......... .......... .
100AB7 .... .......... .......... .......... .......... .......... .......... .......... .......... .......... .......... .......... .......... .......... .......... .......... .......... .......... .......... .......... .......... .......... .......... .......... .
100AB8 .... .......... .......... .......... .......... .......... .......... .......... .......... .......... .......... .......... .......... .......... .......... .......... .......... .......... .......... .......... .......... .T........ .......... .......... .

102AB1 .... .......... .......... .......... .......... .......... .......... .......... .......... .......... .......... .......... .......... .......... .......... .......... .......... .......... .......... .......... .......... .......... .......... .......... .
102AB2 .... .......... .......... .......... .......... .......... .......... .......... .......... .......... .......... .......... .......... .......... .......... .......... .......... .......... .......... .......... .......... .......... .......... .......... .
102AB3 .... .......... .......... .......... .......... .......... .......... .......... .......... .......... .......... .......... .......... .......... .......... .......... .......... .......... .......... .......... .......... .......... .......... .......... .
102AB4 .... .......... .......... .......... .......... .......... .......... .......... .......... .......... .......... .......... .......... .......... .......... .......... .......... .......... .......... .......... .......... .......... .......... .......... .
102AB5 .... .......... .......... .......... .......... .......... .......... .......... .......... .......... .......... .......... .......... .......... .......... .......... .......... .......... .......... .......... .......... .......... .......... .......... .
102AB6 .... .......... .......... .......... .......... .......... .......... .......... .......... .......... .......... .......... .......... .......... .......... .......... .......... .......... .......... .......... .......... .......... .......... .......... .
102AB7 .... .......... .......... .......... .......... .......... .......... .......... .......... .......... .......... .......... .......... .......... .......... .......... .......... .......... .......... .......... .......... .......... .......... .......... .
102AB8 .... .......... .......... .......... .......... .......... .......... .......... .......... ......T... .........G .......... .......... .......... .......... .......... .......... .......... .......... .......... .......... .......... .......... .......... .
102AB9 .... .......... .......... .......... .......... .......... .......... .......... .......... .......... .........G .......... .......... .......... .......... .......... .......... .......... .......... .......... .......... .......... .......... .......... .
102AB10 .... .......... .......... .......... .......... .......... .......G.. .......... .......... .......... .........G .......... .......... .......... .......... .......... .......... .......... .......... .......... .......... .......... .......... .......... .

106AB1 .... .......... .......... .......... .......... .......... .......... .......... .......... .......... .......... .......... .......... .......... .......... .......... .......... .......... .......... .......... .......... .......... .......... .......... .
106AB2 .... .......... .......... .......... .......... .......... .......... .......... .......... .......... .......... .......... .......... .......... .......... .......... .......... .......... .......... .......... .......... .......... .......... .......... .
106AB3 .... .......... .......... .......... .......... .......... .......... .......... .......... .......... .......... .......... .......... .......... .......... .......... .......... .......... .......... .......... .......... .......... .......... .......... .
106AB4 .... .......... .......... .......... .......... .......... .......... .......... .......... .......... .......... .......... .......... .......... .......... .......... .......... .......... .......... .......... .......... .......... .......... .......... .
106AB5 .... .......... .......... .......... .......... .......... .......... .......... .......... .......... .......... .......... .......... .......... .......... .......... .......... .......... .......... .......... .......... .......... .......... .......... .
106AB6 .... .......... .......... .......... .......... .......... .......... .......... .......... .......... .......... .......... .......... .......... .......... .......... .......... .......... .......... .......... .......... ......G... .......... .......... .
106AB7 .... .......... .......... .......... .......... .......... .......... .......... .......... .......... .......... .......... .......... .......... ....A..... .......... .......... .......... .......... .......... .......... .......... .......... .......... .
106AB8 .... .......... .......... .......... .T........ .......... .......... .......... .......... .......... .......... .......... .......... .......... .......... .......... .......... .......... .......... .......... .......... .......... .......... .......... .

109AB1 .... .......... .......... .......... .......... .......... .......... .......... .C........ .......... .......... .......... .......... .......... .......... .......... .......... .......... .......... .......... .......... .......... .......... .......... .
109AB2 .... .......... .......... .......... .......... .......... .......... .......... .......... .......... .......... .......... .......... .......... .......... .......... .......... .......... .......... .......... .......... .......... .......... .......... .
109AB3 .... .......... .......... .......... .......... .......... .......... .......... .......... .......... .......... .......... .......... .......... .......... .......... .......... .......... .......... .......... .......... .......... .......... .......... .
109AB4 .... .......... .......... .......... .......... .......... .......... .......... .......... .......... .......... .......... .......... .......... .......... .......... .......... .......... .......... .......... .......... .......... .......... .......... .
109AB5 .... .......... .......... .......... .......... .......... .......... .......... .......... .......... .......... .......... .......... .......... .......... .......... .......... .......... .......... .......... .......... .......... .......... .......... .
109AB6 .... .......... .......... .......... .......... .......... .......... .......... .......... .......... .......... .......... .......... .......... .......... .......... .......... .......... .......... .......... .......... .......... .......... .......... .
```

```
109AB7 .... .......... .......... .......... .......... .......... .......... .......... .......... .......... .......... .......... .......... .......... .......... .......... .......... .......... .......... .......... .......... .......... .......... .......... .
109AB8 .... .......... .......... .......... .......... .......... .......... .......... .......... .......... .......... .......... .......... .......... .......... ........T. .......... .......... .......... .......... ...T...... .T........ .......... .......... .

111AB1 .... .......... .......... .......... .......... .......... .......... .......... .......... .......... .......... .......... .......... .......... .......... .......... .......... .......... .......... .......... .......... ..T....... .......... .......... .
111AB2 .... .......... .......... .......... ......T... .......... .......... .......... .......... .......... .......... .......... .......... .......... .......... .......... .......... .......... .......... .......... .......... ..T....... .......... .......... .
111AB3 .... .......... .......... .......... .......... .......... .......... .......... .......... .......... .......... .......... .......... .......... .......... .......... .......... .......... .......... .......... .......... ..T....... .......... .......... .
111AB4 .... .......... .......... .......... .......... .......... .......... .......... .......... .......... .......... .......... .......... .......... .......... .......... .......... .......... .......... .......... .......... ..T....... .......... .......... .
111AB5 .... .......... .......... .......... .......... .......... .......... .......... .......... .......... .......... .......... .......... .......... .......... .......... .......... .......... .......... .......... .......... ..T.T..... .......... .......... .
111AB6 .... .......... .......... .......... .......... .......... .......... .......... .......... .......... .......... .......... .......... .......... .......... .......... .......... .......... .......... .......... .......... ..T....... .......... .......... .
111AB7 .... .......... .......... .......... .......... .......... .......... .......... .......... .......... .......... .......... .......... .......... .....A.... .......... .......... .......... .......... .......... .......... ..T.T..... .......... .......... .
111AB8 .... .......... .......... .......... .......... .......... .......... .......... .......... .......... .......... .......... .......... .......... .......... .......... .......... .......... .......... .......... .......... ..T....... .......... .......... .

107AB1 .... .......... .......... .......... .......... .......... .......... .......... .......... .......... .......... .......... .......... .......... .......... .......... .......... .......... .......... .......... .......... ..T....... .......... .......... .
107AB2 .... .......... .......... .......... .......... .......... .......... .......... .......... .......... .......... .......... .......... .......... .......... .......... .......... .......... .......... .......... .......... ..T....... .......... .......... .
107AB3 .... .......... .......... .......... .......... .......... .......... .......... .......... .......... .......... .......... .......... .......... .......... .......... .......... .......... .......... .......... .......... ..T....... .......... .......... .
107AB4 .... .......... .......... .......... .......... .......... .......... .......... .......... .......... .......... .......... .......... .......... .......... .......... .......... .......... .......... .......... .......... ..T....... .......... .......... .
107AB5 .... .......... .......... .......... .......... .......... .......... .......... .......... .......... .......... .......... .......... .......... .......... .......... .......... .......... .......... .......... .......... ..T....... .......... .......... .
107AB6 .... .......... .......... .......... ....T..... .......... .......... .......... .......... .......... .T........ .......... .......... .T........ ...C...... .......... .......... .......... .......... .......... .......... ..T....... .......... .......... .
107AB7 .... .......... .......... .......... ....T..... .......... .......... .......... .......... .......... .......... .......... .......... .......... .......... .......... .......... .......... TTT....... .......... .......... ..T....... .......... .......... .
107AB8 .... .......... .......... .......... .......... .......... .......... .......... .......... .......... .T........ .......... .......... .T........ .......... .......... .......... .......... .......... .......... .......... ..T....... .......... .......... .

121AB1 .... .......... .......... .......... .......... .......... .......... .......... .......... .......... .......... .......... .......... .......... .......... .......... .......... ..C....C. .T........ .......... .......... .......... .......... .......... .
121AB2 .... .......... .......... .......... .......... .......... .......... .......... .......... .......... .......... .......... .......... .......... .......... .......... .......... ..C....C. .T........ .......... .......... .......... .......... .......... .
121AB3 .... .......... .......... .......... .......... .......... .......... .......... .......... .......... .......... .......... .......... .......... .......... .......... .......... ..TT...C. TT........ .......... .......... .......... .......... .......... .
121AB4 .... .......... .......... .......... .......... .......... .......... .......... .......... .......... .......... .......... .......... .......... .......... .......... ......G... ..C....C. .T........ .......... .......... .......... .......... .......... .
121AB5 .... .......... .......... .......... .......... .......... .......... .......... .......... .......... .......... .......... .......... .......... .......... ......TT.. .......... ..C....C. .T........ .......... .......... .......... .......... .......... .
121AB6 .... .......... .......... .......... .......... .......... .......... .......... .......... .......... .......... .......... .......... .......... .......... .......... .......... ..C....C. .T........ .......... .......... .......... .......... .......... .
121AB7 .... .......... .......... .......... .......... .......... .......... .......... .......... .......... ......C... .......... .......... .......... .......... .......... .......... ..C...... .T........ .......... .......... .......... .......... .......... .
121AB8 .... .......... .......... .......... .......... .......... .......... .......... .......... .......... .......... .......... .......... .......... .......... .......... .......... ..C....C. .T..T..... .......... .......... .......... .......... .......... .
121AB9 .... .......... .......... .......... .......... .......... .......... .......... .......... .......... .......... .......... .......... .......... .......... .......... .......... ..C....C. .T........ .......... .......... ....T..... .......... .......... .
121AB10 .... .......... .......... .......... .......... .......... .....T.... .......... .......... .......... .......... ....A..... .......... .......... .......... .......... .......... ..C....C. .T........ .......... .......T.. .......... .......... .......... .

131AB1 .... .......... .......... .......... .......... .......... .......... .......... ..C....... .......... .G........ .......... .......... .......... .......... .......... .......... ......T.C. .......... .......... .......... .......... .......... .......... .
131AB2 .... .......... .......... .......... .......... .......... .......... .......... ..C....... .......... .......... .......... .......... .......... .......... .......... .......... ........C. .......... .......... .......... ......G... .......... .......... .
131AB3 .... .......... .......... .......... .......... .......... .......... .......... ..C....... .......... .......... .......... .......... .......... .......... .......... .......... .....T...T .T........ .......... .......... .......... .......... .......... .
131AB4 .... .......... .......... .......... .......... .......... .......... .......... .......... .......... .......... .......... .......... .......... .......... .......... .......... ........C. .T........ .......... .......... .......... .......... .......... .
131AB5 .... .......... .........T .......... .......... .......... .......... .......... .......... .......... .......... ....A..... .......... .......... .......... .......... .......... ........C. .T........ .......... .......... .......... .......... .......... .
131AB6 .... .......... .......... .......... .......... .......... .......... .......... .......... .......... .......... .......... .......... .......... .......... .......... .......... ........C. .T........ .......... .......... .......... .......... .......... .
131AB7 .... .......... .......... .......... .......... .......... ..T....... .......... .......... .......... .......... .......... .......... .......... .......... .......... .......... ........C. .T........ .......... .......... .......... .......... .......... .
131AB8 .... .......... .......... .......... .......... .......... .......... .......... .......... .......... .......... .......... .......... .......... .......... .......... .......... ........C. .T........ .......... .......... .......... .......... .......... .
131AB9 .... .......... .......... .......... .......G.. .......... .......... .......... .......... ......T... .......... .......... .......... .......... .......... .......... .......... ......T.C. .T........ .......... .......... .......... .......... .......... .
```

```
131AB10 .... .......... .......... .......... .......... .......... .......... .......... .......... .......... .......... .......... .......... .......... T......... .......... .......... .......C.. .T........ .......... .......... .......... .......... .......... .
131AB11 .... .......... .......... .......... .......... .......... .......... .......... .......... .......... .......... .......... .......... .......... .......... .......... .......... .......C.. .T........ .......... .......... .......... .......... .......... .
131AB12 .... .......... .......... .......... .......... .......... .......... .......... .......... .......... .......... .......... .......... .......... .......... .......... .......... .......C.. .T........ .......... .......... .......... .......... .......... .

        1111111111 1111111111 1111111111 1111111111 1111111111 1111111111 1111111111 1111111111 1111111111 1111111111 1111111111 1111111111 1111111111 1111111111 1111111111 1111111111 1111111111 1111111111 1111111111 1111111111 111111111
        6666666666 6666666666 6666666666 6666666666 6666666666 6666666666 6666666666 6666666666 6666666666 6666666666 6666666666 6666666666 6666666666 6666666666 6666666666 6666666666 6666666666 6666666666 6666666666 6666666666 666666666
        2222222222 2222222222 2222222222 2222222222 2222222222 2222222222 2222222222 2222222222 2222222222 2222222223 3333333333 3333333333 3333333333 3333333333 3333333333 3333333333 3333333333 3333333333 3333333333 3333333334 444444444
        0000000001 1111111112 2222222223 3333333334 4444444445 5555555556 6666666667 7777777778 8888888889 9999999990 0000000001 1111111112 2222222223 3333333334 4444444445 5555555556 6666666667 7777777778 8888888889 9999999990 000000000
        1234567890 1234567890 1234567890 1234567890 1234567890 1234567890 1234567890 1234567890 1234567890 1234567890 1234567890 1234567890 1234567890 1234567890 1234567890 1234567890 1234567890 1234567890 1234567890 1234567890 123456789
CRS     CAAGCAAGTA CAGCAATCAA CCCTCAACTA TCACACATCA ACTGCAACTC CAAAGCCACC CCTCACCCAC TAGGATACCA ACAAACCTAC CCACCCTTAA CAGTACATAG TACATAAAGC CATTTACCGT ACATAGCACA TTACAGTCAA ATCCCTTCTC GTCCCCATGG ATGACCCCCC TCAGATAGGG GTCCCTTGAC CACCATCCT
107CD1  .......... .......... ..T....... .......... .......... .......... .......... .......... .......... .......C.. ........G. .......... ......T... ......T... .......... .......... .......... .......... .......... .......... .........
107CD2  .......... .......... ..T....... .......... .......... .......... .......... .......... .......... .......C.. ........G. .......... ......T... .......... .......... .......... .......... .......... .......... .......... .........
107CD3  .......... .......... ..T....... .......... .......... .......... .......... .......... .......... .......C.. ........G. .......... ......T... .......... .......... .......... .......... .......... .......... .......... .........
107CD4  .......... .......... ..T....... .......... .......... .......... .......... .......... .......... .......C.. .......... .......... ......T... .......... .......... .......... .........A .......... .......... .......... .........
107CD5  .......... .......... ..T....... .......... .......... .......... .......... .......... .......... .......C.. ........G. ..T....... ......T... .......... .......... .......... .......... .......... .......... .......... .........
107CD6  .......... .......... ..T....... .......... .......... .......... .......... .......... .........T .......C.. ........G. .......... ......T... .......... .......... .......... .......... .......T.. .......... .......... .........
107CD7  .......... .......... ..T....... .......... .......... .......... .......... .......... .......... ..G.TT.... ........G. .......... ......T... .......... .......... .......... ..T....... .......... .......... .......... .........
107CD8  .......... .......... ..T....... .......... .......... .......... .......... .......... .......... .......C.. ........G. .......... ......T... .......... .......... .......... .......... .......... .......... .......... .........

111CD1  .......... .......... ..T....... .......... .......... .......... .......... .......... .......... .......C.. ........G. .......... ......T... .......... .......... .......... .......... .......... .......... .......... .........
111CD2  .......... .......... ..T....... .......... .......... .......... .......... .......... .......... .......C.. ........G. .......... ......T... .......... .......... .......... .......... .......... .......... .......... .........
111CD3  .......... .......... ..T....... .......... .......... .......... .......... .......... .......... .......C.. ........G. .......... ......T... .......... .......... .......... .......... .......... .......... .......... .........
111CD4  .......... .......... ..T....... .......... .......... .........T .......... .......... .......... .......C.. ........G. .......... ......T... .......... .......... .......... .......... .......... .......... .......... .........
111CD5  .......... .......... ..T....... .......... .......... .......... .......... .......... .......... ......CC.. ........G. .......... ......T... .......... .......... .......... .......... .......... .......... .......... .........
111CD6  .......... .......... ..T....... .......... .......... .......... .......... .......... .......... .......C.. ........G. .......... ......T... ..G....... .......... .......... ..T....... .......... .......... .......... .........
111CD7  .......... .......... ..T....... .......... .T..T..... .........T .......... .......... .......... .......C.. ........G. .......... ......T... .......... .......... .......... .......... .......... .......... .......... .........
111CD8  .......... .......... ..T....... .......... .T..T..... .......... .......... .......... .......... ......CC.. ........G. .......... ......T... .......... .......... .......... .......... .......... .......... .......... .........

117CD1  .......... .......... ..T....... .......... .......... .......... .......... .......... .......... .......... ...C...... .......... .......... .......... .......... .......... .......... .......... .......... .......... .........
117CD2  .......... .......... ..T....... .......... .......... .......... .......... .......... .......... .......... ...C...... .......... .......... .......... .......... .......... .......... .......... .......... .......... .........
117CD3  .......... .......... ..T....... .......... .......... .......... .......... .......... .......... .......... ...C...... .......... .......... .......... .......... .......... .......... .......... .......... .......... .........
117CD4  .......... .......... ..T....... .......... .......... .......... .......... .......... .......... .......... ...C...... .......... .......... .......... .......... .......... .......... .......... .......... .......... .........
117CD5  .......... .......... ..T....... .......... .......... .......... .......... .......... .......... .......... ...C...... .......... .......... .......... .......... .......... .......... .......... .......... .......... .........
117CD6  .......... .......... ..T....... .......... .......... .......... .......... .......... .......... .......... ...C...... .......... .......... .......... .......... .......... .......... .......... .......... .......... .........
117CD7  .......... .......... ..T....... .......... .......... .......... .......... .......... .......... .......... ...C...... .......... .......... .......... .......... .......... .......... .......... .......... .......... .........
117CD8  .......... .......... ..T....... .......... .......... .......... .......... .......... .......... .......... ...C...... .......... .......... .......... .......... .......... .......... .......... .......... .......... .........

120CD1  .......... .......... .......... .......... .......... .......... .......... .......... .......... .......... .......... C......... .......... .......... .......... .......... .......... .......... .......... .......... .........
120CD2  .......... .......... .......... .......... .......... .......... .......... .......... .......... .......... .......... C......... .......... .......... .......... .......... .......... .......... .......... .......... .........
120CD3  .......... .......... .......... .......... .......... .......... .......... .......... .......... .......... .......... C......... .......... .......... .......... .......... .......... .......... .......... .......... .........
```

```
120CD4  .......... .......... .......... .......... .......... .......... .......... .......... .......... .......... .......... C......... .......... .......... .......... .......... .......... .......... .......... .......... .........
120CD5  .......... .......... .......... .......... .......... .......... .......... .......... .......... .......... .......... C......... .......... .......... .......... .......... .......... .......... .......... .......... .........
120CD6  .......... .......... .......... .......... .......... .......... .......... .......... .......... .......... .......... C......... .......... .......... .......... .......... .......... .......... .......... .......... .........
120CD7  .......... .......... .......... .......... .......... .......... .......... .......... .......... .......... .......... C......... .......... .......... .......... .......... .......... .......... .......... .......... .........
120CD8  .......... .......... .......... .......... .......... .......... .......... .......... .......... .......... ...C...... C......... .......... .......... .......... .......... .......... .......... .......... .......... .........

121CD1  .......... .......... .......... .......... .......... .......... .....TT... .......... .......... .......... .......... C......... .......... .......... .......... .......... .......... .......... .......... .......... .........
121CD2  .......... .......... .......... .......... .......... .......... .......... .......... .......... .......... .......... C.......T. .......... .......... .......... .......... .......... ....T..... .......... .......... .........
121CD3  .......... .......... .......... .......... .......... .......... .......... .......... .......... .......... .......... C......... .......... .......... .......... .......... .......... .......... .......... .......... .........
121CD4  .......... .......... .......... .......... .......... .......... .......... .......... .......... .......... .......... C......... .......... .......... .......... .......... .......... .......... .......... .......... .........
121CD5  .......... .......... .......... .......... .......... .......... .......... .......... .......... .......... .......... C........T .......... .......... .......... .......... .......... .......... .......... .......... .........
121CD6  .......... .......... .........G .......... ........C. ........A. ..CT...... ...A...T.. ...G...C.T ..C....... T......... C......... .......... .......... .......... .......... .......... .......... .......... .......... .........
121CD7  .......... .......... .......... .......... .......... .......... .......... .......... .......... ..GT...... .......... C......... .......... .......... .......... .......... .......... .......... .......... .......... .........
121CD8  .......... .......... .......... .......... ....T..... .......... .......... .......T.. .......... .......... .......... C......... .......... .......... .......... .......... .......... .......... .......... .......... .........

127CD1  .......... .......... ..T....... .......... .......... .......... .......... .......... .......... .......C.. ........G. .......... ......T... .......... .......... .......... .......... .......... .......... .......... .........
127CD2  .......... .......... ..T....... .......... .......... .......... .......... .......... .......... .......C.. ........G. .......... ......T... .......... .......... .......... .......... .......... .......... .......... .........
127CD3  .......... .......... ..T....... .......... .......... .......... .......... .......... .......... .......C.. ........G. .......... ......T... .......... .......... .......... .......... .......... .......... .......... .........
127CD4  .......... .......... ..T....... .......... .......... .......... .......... .......... .......... .......C.. ........G. .......... ......T... .......... .......... .......... .......... .......... .......... .......... .........
127CD5  .......... .......... ..T....... .......... .......... .......... .......... .......... .......... .......C.. ........G. .......... ......T... .......... .......... .......... .......... .......... .......... .......... .........
127CD6  .......... .......... ..T....... .......... .......... .......... .......... .......... .......... .......C.. ........G. .......... ......T... .......... .......... .......... .......... .......... .......... .......... .........
127CD7  .......... .......... ..T....... .......... .......... .......... .......... .......... .......... .......C.. .......... .......... ......T... .......... .......... .......... .......... .......... .......... .......... .........
127CD8  .......... .......... ..T....... .......... .......T.. .......... .......... .......... .......... .......... .......... .......... .......... .......... .......... .......... .......... .......... .......... .......... .........

131CD1  .......... .......... .......... .......... .......... .......... .......... .......... .......... .......... .......... .......... .......... .......... .......... .......... .......... .......... .........A .......... .........
131CD2  .......... .......... .......... .......... .......... .......... .......... .......... .......... .......... .......... C......... .......... .......... .......... .......... .......... .......... .........A .......... .........
131CD3  .......... .......... .......... .......... .......... .......... .......... .......... .......... .......... .......... C......... .......... .......... .......... .......... .......... .......... .........A .......... .........
131CD4  .......... .......... .......... .......... .......... .......... .......... .......... .......... .......... .......... C......... .......... .......... .......... .......... .......... .......... .........A .......... .........
131CD5  .......... .......... .......... .......... .......... .......... .......... .......... .....T.... .......... .......... C......... .......... .......... .......... .......... .......... .......... .........A .......... .........
131CD6  .......... .......... .......... .......... .......... .......... .......... .......... .......... .......... .......... C......... .......... .......... .......... .......... .......... .......... .........A .......... .........
131CD7  .......... .......... .......... .......... .......... .......... .......... ...A...... .......... .......... .......... C......... .......... .......... .......... .......... .......... .......... .........A .......... .........
131CD8  .......... .......... .......... .......... .......... .......... .......... .......TT. .......... .......... .....T.... C......... .......... .......... .......... .....C.... .......... .........T .........A .......... .........

135CD1  .......... .......... ..T....... .......... .......... .......... .......... .......... .......... .......C.. ........G. .......... ......T... .......... .......... .......... .......... .......... .......... .......... .........
135CD2  .......... .......... ..T....... .......... .......... .......... .......... .......... .......... .......C.. ........G. .......... ......T... .......... .......... .......... .......... .......... .......... .......... .........
135CD3  .......... .......... ..T....... .......... .......... .......... .......... .......... .......... .......C.. ........G. .......... ......T... .......... .......... .......... .......... .......... .......... .......... .........
135CD4  .......... .......... ..T....... .......... .......... .......... .......... .......... .......... .......C.. ........G. .......... ......T... .......... .......... .......... .......... .......... .......... .......... .........
135CD5  .......... .......... ..T....... .......... .......... .......... .......... .......... .......... .......C.. ........G. .......... ......T... .......... .......... .......... .......... .......... .......... .......... .........
135CD6  .......... .......... ..T....... .......... .......... .......... .......... .......... .......... .......C.. ........G. .......... ......T... .......... .......... .......... .......... .......... .......... .......... .........
135CD7  .......... .......... ..T....... .......... .......... .......... .......... .......... .......... .......C.. ........G. ..T....... ......T... .......... .......... .......... .......... .......... .......... .......... .........
135CD8  .......... .......... ..T....... .......... .......... .......... .......... .......... .........T .......C.. ........G. .......... ......T... .......... .......... .......... .......... ........T. .......... .......... .........
```

```
109CD1   .......... .......... .......... .......... .......... .......... .......... .......... .......... .......C.. .......... .......... ......T... .......... .......... .......... .......... .......... .......... .......... .........
109CD2   .......... .......... .......... .......... .......... .......... .......... .......... .......... .......C.. .......... .......... ......T... .......... .......... .......... .......... .......... .......... .......... .........
109CD3   .......... .......... .......... .......... .......... .......... .......... .......... .......... .......C.. .......... .......... ......T... .......... .......... .......... .......... .......... .......... .......... .........
109CD4   .......... .......... .......... .......... .......... .......... .......... .......... .......... .......C.. .......... .......... ......T... .......... .......... .......... .......... .......... .......... .......... .........
109CD5   .......... .......... .......... .......... .......... .......... .......... .......... .......... .......C.. .......... .......... ......T... .......... .......... .......... .......... .......... .......... .......... .........
109CD6   .......... .......... .......... .......... .......... .......... .......... .......... .......... .......C.. .......... .......... ......T... .......... .......... .......... .......... .......... .......... .......... .........
109CD7   .......... .......... .......... .......... .......... .......... .......... .......... .......... .......C.. .......... .......... ......T... .......... .......... .......... .......... .......... .......... .......... .........
109CD8   .......... .......... .......... .......... .........T .......... .......T.. .......... .......... .......C.. .......... .......... ......T... .......... .......... .......... ..TT...... .......... .......... .......... .........

100CD1   .......... .......... .......... .......... .......... .......... .......... .......... .......... .......C.. .......... .......... ......T... .......... .......... .......... .......... .......... .......... .......... .........
100CD2   .......... .......... .......... .......... .......... .......... .......... .......... .......... .......C.. .......... .......... ......T... .......... .......... .......... .......... .......... .......... .......... .........
100CD3   .......... .......... .......... .......... .......... .......... .......... .......... .......... .......C.. .......... .......... ......T... .......... .......... .......... .......... .......... .......... .......... .........
100CD4   .......... .......... .......... .......... .......... .......... .......... .......... .......... .......C.. .......... .......... ......T... .......... .......... .......... .......... .......... .......... .......... .........
100CD5   .......... .......... .......... .......... .......... .......... .......... .......... .......... .......C.. .......... .......... ......T... .......... .......... .......... .......... .......... .......... .......... .........
100CD6   .......... .......... .......... .......... .......... .......... .......... .......... .......... .......C.. .......... .......... ......T... .......... .......... .......... .......... .......... .......... .......... .........
100CD7   .......... .......... .......... .......... .......... .......... .......... .......... .......... .......C.. .......... .......... ......T... .......... .......... .......... .......... .......... .......... .......... .........
100CD8   .......... .......... .......... .......... .......... .......... .T........ .......... .......... .......C.. .......... .......... ......T... .......... .......... .......... .......... .......... .......... .......... .........

110CD1   .......... .......... .......... .......... .......... .......... .......... .......... .......... .......C.. .......... .......... ......T... .......... .......... .......... .......... .......... .......... .......... .........
110CD2   .......... .......... .......... .......... .......... .......... .......... .......... .......... .......C.. .......... .......... ......T... .......... .......... .......... .......... .......... .......... .......... .........
110CD3   .......... .......... .......... .......... .......... .......... .......... .......... .......... .......C.. .......... .......... ......T... .......... .......... .......... .......... .......... .......... .......... .........
110CD4   .......... .......... .......... .......... .......... .......... .......... .......... .......... .......C.. .......... .......... ......T... .......... .......... .......... .......... .......... .......... .......... .........
110CD5   .......... .......... .......... .......... .......... .......... .......... .......... .......... .......C.. .......... .......... ......T... .......... .......... .......... .......... .......... .......... .......... .........
110CD6   .......... .......... .......... .......... .......... .......... .......... .......... .......... .......C.. .......... .......... ......T... .......... .......... .......... .......... .......... .......... .......... .........
110CD7   .......... .......... .......... .......... .......... .......... .......... .......... .......... .......C.. .......... .......... ......T... .......... .......... .......... .......... .......... .......... .......... .........
110CD8   .......... .......... .......... .......... .......... .......... .......... .......... .......... .......C.. .......... .......... ......T... .......... .......... .......... .......... .......... .......... .......... .........
110CD9   .......... .......... .......... .......... .......... .......... .......... .......... .......... .......C.. ..A....... .......... ......T... .......... .......... .......... .......... .......... .......... .......... .........
110CD10  .......... .......... .......... .......... .......... .......... .......... .......... .......... .......C.. ..A....... .......... ......T... .......... .......... .......... .......... .......... .......... .......... .........

106CD1   .......... .......... .......... .......... .......... .......... .......... .......... .......... .......C.. .......... .......... ......T... .......... .......... .......... .......... .......... .......... .......... .........
106CD2   .......... .......... .......... .......... .......... .......... .......... .......... .......... .......C.. .......... .......... ......T... .......... .......... .......... .......... .......... .......... .......... .........
106CD3   .......... .......... .......... .......... .......... .......... .......... .......... .......... .......C.. .......... .......... ......T... .......... .......... .......... .......... .......... .......... .......... .........
106CD4   .......... .......... .......... .......... .......... .......... .......... .......... .......... .......C.. .......... .......... ......T... .......... .......... .......... .......... .......... .......... .......... .........
106CD5   .......... .......... .......... .......... .......... .......... .......... .......... .......... .......C.. .......... .......... ......T... .......... .......... .......... .......... .......... .......... .......... .........
106CD6   .......... .......... .......... .......... .......... .......... .......... .......... .......... .......C.. .......... .......... ......T... .......... .......... .......... .......... .....T.... .......... .......... .........
106CD7   .......... .......... .......... .......... .......... .......... .......... .......... .......... .......C.. .......... .......... ......T... .......... .......... .......... .......... .......... .......... .......... .........
106CD8   .......... .......... .......... .......... .......... ........T. .......... .......... .......... .......C.. .......... .......... T.....T... .......... .......... .......... .......... .......... .......... .......... .........

115CD1   .......... .......... .......... .......... .......... .......... .......... .......... .......... .......C.. .......... .......... ......T... .......... .......... .......... .......... .......... .......... .......... .........
115CD2   .......... .......... .......... .......... .......... .......... .......... .......... .......... .......C.. .......... .......... ......T... .......... .......... .......... .......... .......... .......... .......... .........
```

115CD3 ....C.. ....T... ....
115CD4 ....T. ....C.. ....T... ....
115CD5 ....T. ....C.. ....TT.. ....T..... ....
115CD6 ....C.. ....T... ....
115CD7 ....C.. ....T... ....
115CD8 ....T. ....C.. ....T... ....T...... ....T...T. ....

119CD1 ....C...... .... C......... ....A ....
119CD2 ....C...... .... C......... ....A ....
119CD3 ....C...... .... C......... ....A ....
119CD4 ....C...... .... C......... ....A ....
119CD5 ....C...... .... C......... ....A ....
119CD6 ....C...... .... C......... ....A ....
119CD7 ....C...... .... C......... ....A ....
119CD8 .... .T.C...... .... C......... ....A ....

128CD1 ....T ....
128CD2 ....T ....
128CD3 ....T ....
128CD4 ....T ....
128CD5 ....T ....
128CD6 ....T ....
128CD7 ....T ....
128CD8 ....T ....

132CD1 ....C.. ....T... ....
132CD2 ....C.. ....T... ....
132CD3 ....C.. ....T... ....
132CD4 ....C.. ....T... ....
132CD5 ....C.. ....T... ....
132CD6 ....C.. ....T... ....
132CD7 ....T. ....C.. ....T... ...T...... ....
132CD8 ....T. ....C.. ....T... ...T...... ....

136CD1 ....C.. ....T... ....
136CD2 ....C.. ....T... ....
136CD3 ....C.. ....T... ....
136CD4 ....C.. ....T... ....
136CD5 ....T... ....
136CD6 ....C.. ....T... ....

```
136CD7 .......... .......... .......... .......... .......... .......... .......... .......... .......... .......C.. .......... .......... ......T... .......... .......... .......... .......... .......... .......... .......... .........
136CD8 .......... .......... .......... .......... .......... .......... .......... .......... .......... .......C.. .......... .......... ......T... .......... .......... ...T.TT... .......... .......... .......... .......... .........

138CD1 .......... .......... .......... .......... .......... .......... .......... .......... .......... .......C.. .......... .......... ......T... .......... .......... .......... .......... .......... .......... .......... .........
138CD2 .......... .......... .......... .......... .......... .......... .......... .......... .......... .......C.. .......... .......... ......T... .......... .......... .......... .......... .......... .......... .......... .........
138CD3 .......... .......... .......... .......... .......... .......... .......... .......... .......... .......C.. .......... .......... ......T... .......... .......... .......... .......... .......... .......... .......... .........
138CD4 .......... .......... .......... .......... .......... .......... .......... .......... .......... .......C.. .......... .......... ......T... .......... .......... .......... .......... .......... .......... .......... .........
138CD5 .......... .......... .......... .......... .......... .......... .......... .......... .......... .......C.. .......... .......... ......T... .......... .......... .......... .......... .......... .......... .......... .........
138CD6 .......... .......... .......... .......... .......... .......... .......... .......... .......... .......C.. .......... .......... ......T... .......... .......... .......... .......... .......... .......... .......... .........
138CD7 .......... .......... .......... .......... .......... .......... .......... .......... .......... .......C.. .......... .......... ......T... ........T. .......... .......... .......... .......... .......... .......... .........
138CD8 .......... .......... .......... .......... .......... .......... .......... .......... .......... .......C.. .......... .......... ......T... ........T. .......... .......... .......... .......... .......... .......... .........

139CD1 .......... .......... .......... .......... .......... .......... .......... .......... .......... .......C.. .......... .......... ......T... .......... .......... .......... .......... .......... .......... .......... .........
139CD2 .......... .......... .......... .......... .......... .......... .......... .......... .......... .......C.. .......... .......... ......T... .......... .......... .......... .......... .......... .......... .......... .........
139CD3 .......... .......... .......... .......... .......... .......... .......... .......... .......... .......C.. .......... .......... ......T... .......... .......... .......... .......... .......... .......... .......... .........
139CD4 .......... .......... .......... .......... .......... .......... .......... .......... .......... .......C.. .......... .......... ......T... .......... .......... .......... .......... .......... .......... .......... .........
139CD5 .......... .......... .......... .......... .......... .......... .......... .......... .......... .......C.. .......... .......... ......T... .......... .......... .......... .......... .......... .......... .......... .........
139CD6 .......... .......... .......... .......... .......... .......... .......... .......... .......... .......... .......... .......... ......T... .......... .......... .......... .......... .......... .......... .......... .........
139CD7 .......... .......... .......... .......... .......... .......... .......... .......... .......... .......C.. ..A.....G .......... ......T... .......... .......... .......... .......... .......... .......... .......... .........
139CD8 .......... .......... .......... .......... .......... .......... .......... .......... .......... .......C.. ..A.....G .......... ......T... .......... .......... .......... .......... .......... .......... .......... .........
```

Fig A1. The results of clone sequencing
