# Supplementary material for: Evidence that a West-East admixed population lived in the Tarim Basin as early as the early Bronze Age
Source: BMC Biol. 2010 Feb 17;8:15. doi: 10.1186/1741-7007-8-15 (PMC2838831; doi:10.1186/1741-7007-8-15)
Supplement: Additional file 3 — Table S2. Estimated frequencies of mitochondrial DNA haplogroup C in modern populations. [file 1741-7007-8-15-S3.DOC]

| Location(population size) | Populations(size/C size) | The frequency of C | References |
| --- | --- | --- | --- |
| Altai region(280) |  | 21.79 |  |
|  | Altaian1(98/26) |  | 1 |
|  | Tubalar(72/14) |  | 2 |
|  | Altaian2(110/21) |  | 3 |
| Tuva Republic(327) |  | 47.71 |  |
|  | Tuvinians1(36/13) |  | 4 |
|  | Tuvinians2(95/41) |  | 2 |
|  | Todjin(48/23) |  | 3 |
|  | Tofalar1(58/36) |  | 3 |
|  | Tuvinians3(90/43) |  | 3 |
| Baikal lake(282) |  | 22.7 |  |
|  | Buryat1(126/20) |  | 5 |
|  | Buryat2(40/8) |  | 4 |
|  | Buryat3(25/10) |  | 2 |
|  | Buryat4(91/26) |  | 3 |
|  | Sojots(30/6) |  | 3 |
| Southeast Siberia (222) |  | 11.26 |  |
|  | Udegeys(46/8) |  | 6 |
|  | Ulchi(87/12) |  | 2 |
|  | Negidal(33/5) |  | 2 |
|  | Nivkhs(56/0) |  | 2 |
| Northeast Siberia(316) |  | 24.05 |  |
|  | Koryaks(155/60) |  | 2 |
|  | Itlmen(47/7) |  | 2 |
|  | Chukchi(66/7) |  | 2 |
|  | Eskimos(78/2) |  | 2 |
| Central Siberia(231) |  | 52.8 |  |
|  | Yakut(117/46) |  | 5 |
|  | Evenk(71/51) |  | 2 |
|  | Evens(43/25) |  | 6 |
| West Siberia |  | 17.3 |  |
|  | Mansi(98/17) |  | 2 |
| North Siberia(87) |  | 28.7 |  |
|  | Nganasan(49/19) |  | 6 |
|  | Ket(38/6) |  | 2 |
| Mongolia(192) |  | 16.15 |  |
|  | Mongolia1(103/15) |  | 7 |
|  | Mongolia2(89/16) |  | 8 |
| Inner-mogolia (232) |  | 11.21 | 9 |
|  | Daur(45/3) |  |  |
|  | Oroqen(44/11) |  |  |
|  | Mongolian(48/3) |  |  |
|  | Evenki(47/9) |  |  |
|  | korean(48/0) |  |  |
| Xinjiang(299) |  | 6.02 | 10 |
|  | Mongolian(49/5) |  |  |
|  | Hui(45/1) |  |  |
|  | Kazak(53/7) |  |  |
|  | Uighur(47/4) |  |  |
|  | Han(47/0) |  |  |
|  | Uzbek(58/1) |  |  |
| Qinghai (50) |  | 6 | 11 |
|  | Tu(35) |  |  |
|  | Mongolian(15) |  |  |
| Central Asia(391) |  | 8.18 |  |
|  | Tukmenastan(73/6) |  | 12 |
|  | Uzbkstan(42/1) |  | 12 |
|  | Tajikistan(44/8) |  | 12 |
|  | Central Asia2(232/17) |  | 13 |

Table S2. Estimated frequencies of mtDNA haplogroup C in modern populations.

References

1. Phillips-Krawczak C, Devor E, Zlojutro M, Moffat-Wilson K, Crawford MH: **MtDNA variation in the Altai-Kizhi population of southern Siberia: a synthesis of genetic variation.** *Human Biology* 2006, **78**(4): 477-494.
2. [Starikovskaya EB](http://www.ncbi.nlm.nih.gov/sites/entrez?Db=pubmed&Cmd=Search&Term="Starikovskaya EB"%5BAuthor%5D&itool=EntrezSystem2.PEntrez.Pubmed.Pubmed_ResultsPanel.Pubmed_DiscoveryPanel.Pubmed_RVAbstractPlus), [Sukernik RI](http://www.ncbi.nlm.nih.gov/sites/entrez?Db=pubmed&Cmd=Search&Term="Sukernik RI"%5BAuthor%5D&itool=EntrezSystem2.PEntrez.Pubmed.Pubmed_ResultsPanel.Pubmed_DiscoveryPanel.Pubmed_RVAbstractPlus), [Derbeneva OA](http://www.ncbi.nlm.nih.gov/sites/entrez?Db=pubmed&Cmd=Search&Term="Derbeneva OA"%5BAuthor%5D&itool=EntrezSystem2.PEntrez.Pubmed.Pubmed_ResultsPanel.Pubmed_DiscoveryPanel.Pubmed_RVAbstractPlus), [Volodko NV](http://www.ncbi.nlm.nih.gov/sites/entrez?Db=pubmed&Cmd=Search&Term="Volodko NV"%5BAuthor%5D&itool=EntrezSystem2.PEntrez.Pubmed.Pubmed_ResultsPanel.Pubmed_DiscoveryPanel.Pubmed_RVAbstractPlus), [Ruiz-Pesini E](http://www.ncbi.nlm.nih.gov/sites/entrez?Db=pubmed&Cmd=Search&Term="Ruiz-Pesini E"%5BAuthor%5D&itool=EntrezSystem2.PEntrez.Pubmed.Pubmed_ResultsPanel.Pubmed_DiscoveryPanel.Pubmed_RVAbstractPlus), [Torroni A](http://www.ncbi.nlm.nih.gov/sites/entrez?Db=pubmed&Cmd=Search&Term="Torroni A"%5BAuthor%5D&itool=EntrezSystem2.PEntrez.Pubmed.Pubmed_ResultsPanel.Pubmed_DiscoveryPanel.Pubmed_RVAbstractPlus), [Brown MD](http://www.ncbi.nlm.nih.gov/sites/entrez?Db=pubmed&Cmd=Search&Term="Brown MD"%5BAuthor%5D&itool=EntrezSystem2.PEntrez.Pubmed.Pubmed_ResultsPanel.Pubmed_DiscoveryPanel.Pubmed_RVAbstractPlus), [Lott MT](http://www.ncbi.nlm.nih.gov/sites/entrez?Db=pubmed&Cmd=Search&Term="Lott MT"%5BAuthor%5D&itool=EntrezSystem2.PEntrez.Pubmed.Pubmed_ResultsPanel.Pubmed_DiscoveryPanel.Pubmed_RVAbstractPlus), [Hosseini SH](http://www.ncbi.nlm.nih.gov/sites/entrez?Db=pubmed&Cmd=Search&Term="Hosseini SH"%5BAuthor%5D&itool=EntrezSystem2.PEntrez.Pubmed.Pubmed_ResultsPanel.Pubmed_DiscoveryPanel.Pubmed_RVAbstractPlus), [Huoponen K](http://www.ncbi.nlm.nih.gov/sites/entrez?Db=pubmed&Cmd=Search&Term="Huoponen K"%5BAuthor%5D&itool=EntrezSystem2.PEntrez.Pubmed.Pubmed_ResultsPanel.Pubmed_DiscoveryPanel.Pubmed_RVAbstractPlus), [Wallace DC](http://www.ncbi.nlm.nih.gov/sites/entrez?Db=pubmed&Cmd=Search&Term="Wallace DC"%5BAuthor%5D&itool=EntrezSystem2.PEntrez.Pubmed.Pubmed_ResultsPanel.Pubmed_DiscoveryPanel.Pubmed_RVAbstractPlus): **Mitochondrial DNA diversity in indigenous populations of the southern extent of Siberia, and the origins of native American haplogroups**. *Annals of Human Genetics* 2005, **69**:67–89.
3. Derenko MV, Grzybowski T, Malyarchuk BA, Dambueva IK, Denisova GA, Czarny J, Dorzhu CM, Kakpakov VT, Miścicka-Sliwka D, Woźniak M, Zakharov I A: **Diversity of mitochondrial DNA lineages in south Siberia**. *Annals of Human Genetic s*2003, **67**:391–411.
4. Derenko MV, Malyarchuk BA, Dambueva IK, Shaikhaev GO, Dorzhu CM, Nimaev DD, Zakharov IA: **Mitochondrial DNA variation in two south Siberian aboriginal populations: implications for the genetic history of North Asi**a. *Human Biology* 2000, **72**(6) 945-973.
5. Pakendorf B, Wiebe V, Tarskaia L A, Spitsyn VA, Soodyall H, Rodewald A, Stoneking M: **Mitochondrial DNA evidence for admixed origins of central Siberian population**. [*Am J Phys Anthropol* 2003](javascript:AL_get(this, 'jour', 'Am J Phys Anthropol.');), **120**:211–224.
6. Torroni A, Sukernik R1, Schurr Te G, Starikovskaya YB, Cabell M F, Crawford MH, Comuzzie AG, Wallace DC: **MtDNA variation of aboriginal Siberians reveals distinct genetic affinities with native Americans**. *Am.J. Hum. Genet* 1993a, **53**:591-608.
7. Kolman CJ, Sambuughin N, Bermingham E: **Mitochondrial DNA analysis of Mongolian populations and implications for the origin of New Word founders.** *Genetics* 1996, **142**(4):1321-1334.
8. Gokcumen O, Dulik M C, Pai AA, Zhadanov SI, Rubinstein S, Osipova LP, Andreenkov OV, Tabikhanova LE, Gubina MA, Labuda D, Schurr TG: **Genetic variation in the enigmatic Altaian Kazakhs of south-central Russia: insights intoTurkic population history.** *Am J Phys Anthropol* 2008,**136**:278–293
9. Kong QP, Yao YG, Liu M, Shen SP, Chen C, Zhu CL, Palanichamy MG, Zhang YP: **Mitochondrial DNA sequence polymorphisms of five ethnic populations from northern China**. *Hum. Genet* 2003a, **113**:391–405.
10. Yao YG, Kong QP, Wang CY, Zhu CL, Zhang YP: **Different matrilineal contributions to genetic structure of ethnic groups in the Silk Road region in China**. *Mol. Biol. Evol*2004, **21**(12):2265–2280.
11. [Yao YG](http://www.ncbi.nlm.nih.gov/sites/entrez?Db=pubmed&Cmd=Search&Term="Yao YG"%5BAuthor%5D&itool=EntrezSystem2.PEntrez.Pubmed.Pubmed_ResultsPanel.Pubmed_DiscoveryPanel.Pubmed_RVAbstractPlus), [Nie L](http://www.ncbi.nlm.nih.gov/sites/entrez?Db=pubmed&Cmd=Search&Term="Nie L"%5BAuthor%5D&itool=EntrezSystem2.PEntrez.Pubmed.Pubmed_ResultsPanel.Pubmed_DiscoveryPanel.Pubmed_RVAbstractPlus), [Harpending H](http://www.ncbi.nlm.nih.gov/sites/entrez?Db=pubmed&Cmd=Search&Term="Harpending H"%5BAuthor%5D&itool=EntrezSystem2.PEntrez.Pubmed.Pubmed_ResultsPanel.Pubmed_DiscoveryPanel.Pubmed_RVAbstractPlus), [Fu YX](http://www.ncbi.nlm.nih.gov/sites/entrez?Db=pubmed&Cmd=Search&Term="Fu YX"%5BAuthor%5D&itool=EntrezSystem2.PEntrez.Pubmed.Pubmed_ResultsPanel.Pubmed_DiscoveryPanel.Pubmed_RVAbstractPlus), [Yuan ZG](http://www.ncbi.nlm.nih.gov/sites/entrez?Db=pubmed&Cmd=Search&Term="Yuan ZG"%5BAuthor%5D&itool=EntrezSystem2.PEntrez.Pubmed.Pubmed_ResultsPanel.Pubmed_DiscoveryPanel.Pubmed_RVAbstractPlus), [Zhang YP](http://www.ncbi.nlm.nih.gov/sites/entrez?Db=pubmed&Cmd=Search&Term="Zhang YP"%5BAuthor%5D&itool=EntrezSystem2.PEntrez.Pubmed.Pubmed_ResultsPanel.Pubmed_DiscoveryPanel.Pubmed_RVAbstractPlus): **Genetic relationship of Chinese ethnic populations revealed by mtDNA sequence diversity**. [*Am J Phys Anthropol* 2002](javascript:AL_get(this, 'jour', 'Am J Phys Anthropol.');), **118**(1):63-76
12. Quintana-Murci L, Chaix Rl, Wells RS, Behar D M, Sayar H, Scozzari R, Rengo C, Al-Zahery N, Semino O,. Santachiara-Benerecetti AS, Coppa A, Ayub Q, Mohyuddin A, Tyler-Smith C, Mehd SQ, Torroni A, McElreavey K: **Where West meets East: the complex mtDNA landscape of the southwest and central Asian corridor**. *Am. J. Hum. Genet* 2004,**74**:827–845,
13. Comas D, Plaza S., Wells RS, Yuldaseva N, Lao O, Calafell F, Bertranpetit J: **Admixture, migrations, and dispersals in Central Asia: evidence from maternal DNA lineages**. *Eur. J. Hum. Genet* 2004, **12**:495–504.
